# Longitudinal Study of the Occurrence of Usutu Virus and West Nile Virus Infections in Birds in a Zoological Garden in Northern Germany

**DOI:** 10.3390/pathogens12060753

**Published:** 2023-05-23

**Authors:** Felicitas Bergmann, Martina Schmoock-Wellhausen, Christine Fast, Cora M. Holicki, Friederike Michel, Patrick Wysocki, Balal Sadeghi, Martin H. Groschup, Ute Ziegler

**Affiliations:** 1 Friedrich-Loeffler-Institut, Federal Research Institute for Animal Health, Institute of Novel and Emerging Infectious Disease, Südufer 10, 17493 Greifswald-Insel Riems, Germany; felicitas.bergmann@fli.de (F.B.); christine.fast@fli.de (C.F.); cora.holicki@fli.de (C.M.H.); friederike.michel@lua.sms.sachsen.de (F.M.); balal.sadeghi@fli.de (B.S.); martin.groschup@fli.de (M.H.G.); 2 Wildpark Schwarze Berge GmbH & Co. KG, Am Wildpark 1, 21224 Rosengarten, Germany; fachtierarzt@yahoo.de; 3 Tiermedizin am Rothenbaum, Rothenbaumchaussee 195, 20149 Hamburg, Germany; 4 Friedrich-Loeffler-Institut, Federal Research Institute for Animal Health, Institute of Epidemiology, Südufer 10, 17493 Greifswald-Insel Riems, Germany; patrick.wysocki@fli.de

**Keywords:** USUV, WNV, bird, zoo, monitoring, surveillance

## Abstract

Usutu virus (USUV) and West Nile virus (WNV) are known to cause diseases and mortalities in bird populations. Since 2010/2011, USUV has circulated in Germany and spread nationwide, while WNV was only introduced into East Germany in 2018. The zoological garden investigated is located in Northern Germany, where USUV infections in wild birds have been detected for several years. In this longitudinal study conducted over a four-year period, zoo birds were sampled biannually and screened for molecular and serological evidence of USUV and WNV. USUV genomes were detected in eight of the sampled birds and whole-genome sequences revealed the circulation of USUV lineages Europe 3 and Africa 3. Of the eight birds infected with USUV during the study period, four died after the infection, while four survived without displaying clinical signs. Furthermore, in a few of the birds, a USUV (re-)infection was confirmed on a serological level with three birds producing USUV-neutralizing antibodies (nAbs) over a period of four years. Nonetheless, in two birds sampled throughout this longitudinal study, neither a USUV nor a WNV infection was evident. In 2022, WNV nAbs were detected for the first time in a juvenile zoo bird, indicating the introduction of the virus into this region.

## 1. Introduction

Usutu virus (USUV) and West Nile virus (WNV) (family *Flaviviridae*) belong to the Japanese encephalitis antigenic complex, are well-established in Europe and Germany, and circulate in an enzootic cycle between ornithophilic mosquitoes as vectors and birds as amplifying hosts. Due to their high level of viremia and their capability of infecting mosquitoes, birds serve as reservoir hosts. Certain infected bird species may develop signs of disease [1,2]. In Europe in particular, USUV caused high mortality rates in several avian species. Mammals, such as humans and horses, are considered dead-end hosts [3].

In Germany, wild birds have been monitored for several years in the frame of surveillance programs to track arbovirus infections [4,5]. However, zoological gardens also provide valuable opportunities to monitor viral infections. They are usually located near urban areas, enabling close contact between wildlife, arthropods, and humans. Furthermore, in zoological facilities different animal species from around the world are housed location-based and often for life. They are kept in close proximity to each other and are clinically monitored by animal caretakers and veterinarians, as it is of particular importance to detect zoonotic diseases at early stages to ensure the biosafety of other animals and of visitors. The detection of pathogens in sedentary birds indicates the presence of a pathogen in a defined area [6], as opposed to detection in migrating wild birds stopping over in Germany. Zoo birds can serve as ideal early-warning systems for flaviviruses and have therefore in the past years been gradually integrated into German surveillance programs [4,7].

In 2001, USUV infections, associated with severe bird mortalities, were detected in Austria for the first time in Europe [8]. In subsequent years, high USUV mortality rates were observed in zoo birds in several European countries, e.g., in Austria [8,9], Italy [10], Switzerland [9,11], and Hungary [9]. In Germany, however, the first infected mosquito was only found in 2010 [12]. In 2011, mass mortalities were observed among birds, especially blackbirds (*Turdus merula*) and various owl species, as well as in other bird species from zoological gardens [13]. From then on, the virus continued to cause deaths in the avifauna [4,5]. In contrast, WNV has spread continuously across Europe since the late 1990s, with reported infections in humans, horses, and birds [1]. In Germany, WNV was detected for the first time in a great grey owl originating from Zoo Halle, East Germany, in 2018 [14]. Since its introduction, WNV has gradually continued to spread throughout eastern Germany [4]. Since 2019, autochthonous human cases have been registered, with the first fatal outcome also from an endemic region in East Germany [7].

This study focused on the serological and molecular monitoring of birds in the zoological garden Wildpark Schwarze Berge, located in Lower Saxony. USUV has been circulating in Northern Germany for quite some time [4,5,15], while WNV was only detected in 2019 and 2022 with infections in wild birds and a horse in Hamburg [16]. In this longitudinal study, the incidence of flaviviruses, specifically WNV and USUV, was examined over a four-year period in a long-standing bird population. The objective was to sample a wide range of bird species in order to investigate the presence of USUV-neutralizing antibodies (nAbs) in light of reported deaths in wild birds in 2018 [15]. In this context, this study aimed to examine on the one hand how long flavivirus-specific nAbs remained present in individual birds, and on the other the detection of infections in birds. The paramount goal in the future is the protection of valuable individual birds as well as the prevention of new introductions into zoos.

## 2. Materials and Methods

### 2.1. Sample Collection

**Figure 1:**
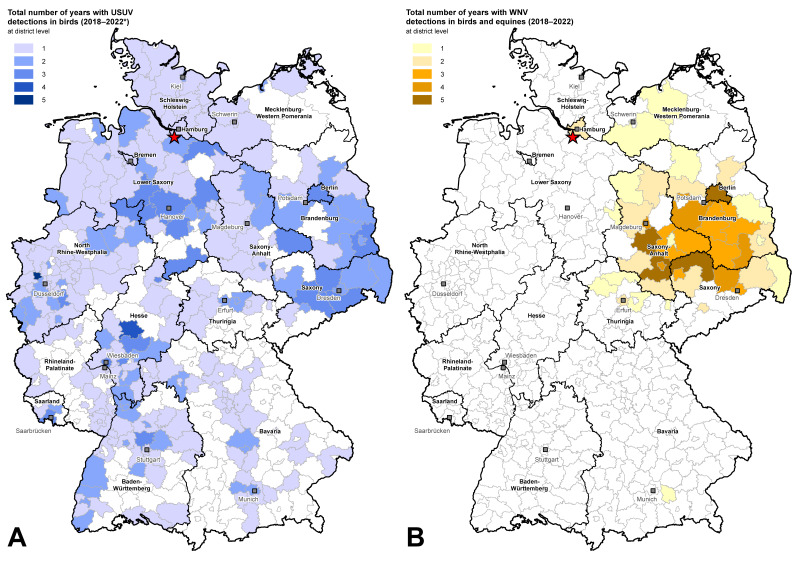
Circulation of USUV and WNV for several years in birds in Germany at district level. The localization of Wildpark Schwarze Berge is marked with a red star on the map. (**A**) Total number of detected USUV genome-positive birds in Germany from 2017 to 2021. Blue-shaded districts were affected by USUV—the darker the color, the more years in which the virus was detected. (**B**) Number of detected WNV-infected birds (RNA) and horses (RNA or IgM-antibodies) in Germany from 2018 to 2022. Orange-shaded districts were affected by WNV—the darker the color the more years in which the virus was detected. * All samples were recorded from a nationwide database [4] which was updated on 13 March 2023.

This study took place at the zoological facility Wildpark Schwarze Berge in Lower Saxony (Figure 1). The zoo extends over a hilly landscape with natural and artificial ponds. Most of the sampled birds were zoo birds aside from a few wild birds that were found injured close to the zoological facility. Between October 2018 and October 2022 (a period of four years), a total of 92 specimens belonging to seven bird orders (*Anseriformes*, *Ciconiiformes*, *Coraciiformes*, *Galliformes*, *Gruiformes*, *Passeriformes*, and *Strigiformes*) were part of this study. The animals were sampled during routine medical examinations. Samples were collected and analyzed from the same birds for several consecutive years in order to study the temporal course of an infection and antibody development. Unfortunately, it was not possible to sample all birds over the entire time period due to population dynamics (birds being sold or purchased or deceased) (Table S1). To minimize stress and avoid harming the valuable animals, care was taken to only restrain the animals for blood collection for the shortest time possible. Approximately 1.0 mL of whole blood was collected in serum separator medium (S-Monoject, Sarstedt, Germany). For this purpose, metatarsal, jugular wing, or ulnar vein puncture was performed by the resident veterinarian. Blood was drawn biannually: at the beginning of the mosquito season in spring (S) (except 2022) and at the end of the season in autumn (A). The samples were centrifuged and the serum (−20 °C) was stored separately from the blood coagulum (−70 °C) until shipment on ice to the Friedrich-Loeffler-Institut (FLI). In addition to the monitoring of the blood, necropsy of dead zoo birds was performed at the national reference laboratory for WNV at the FLI.

### 2.2. Molecular Investigation

Viral RNA from blood coagulum and/or tissue material (mostly brain, liver, spleen, and heart) was extracted using the RNeasy Mini Kit (Qiagen, Hilden, Germany) according to the manufacturer’s instructions. Reverse transcription quantitative real-time PCR (RT-qPCR) was performed by using an assay specific for WNV [17] and for USUV [12]. RT-qPCR for WNV used specific 5′NTR primers and probes [17], with RT-qPCR for USUV focusing on the gene encoding the non-structural protein 1 [12]. To confirm positive USUV-RT-qPCR results an additional specific assay for USUV was performed targeting the NS5 gene [18]. An internal control RNA (IC RNA) containing 2 × 10^5^ copies/µL was extracted together with all samples and included as a duplex RT-qPCR [19]. To ensure the RT-qPCR’s success, negative controls and positive controls were run in parallel. In general, quantification cycle (Ct) values ≤ 37 were regarded as positive and >37 as negative.

### 2.3. Whole Genome Sequencing and Phylogenetic Analysis

All samples positive for USUV with Ct-values ≤32 were sequenced using the MinION (Oxford Nanopore Technologies, Oxford Science Park, Oxford, the United Kingdom (ONT)) with a prior amplicon-enrichment step, and data analysis was performed as described by Holicki et al. 2022 [20]. In short, complementary DNA was synthesized using the multiplex PCR (SuperScript IV First-Strand Synthesis System; Cat. no. 18091050; Invitrogen by Thermo Fisher Scientific, Darmstadt, Germany) with random primers (Invitrogen) [21]. This was followed by an amplification step with 32 primer pairs in two separate reactions using the AccuPrime Taq DNA Polymerase High Fidelity (Cat. no. 12346-086; Invitrogen) [22]. A purification step was performed (Agencourt AMPure XP beads; Agencourt) followed by the preparation of barcoding and ligation mixes using NEBNext Ultra II End Repair/dA-Tailing Module, NEBNext Ultra II Ligation Module, and NEBNext Quick Ligation Module (New England Biolabs, Ipswich, MA, USA), 1D Native Barcoding Genomic DNA Kit (with EXP-NBD104 and SQK-LSK109; ONT), and Flow Cell Priming Kit (EXP-FLP002; ONT) according to the manufacturer’s instructions. The MinION MK1c instrument (ONT) was used with a Spot-ON flow cells (R9.4.1; ONT).

In the MK1C sequencer, basecalling, demultiplexing, and adaptor trimming were performed using Guppy v4.5.4. The consensus sequence was then generated using Minimap2 and mapped to reference genome [23]. BLASTn with default settings was used for the analysis of the consensus sequence [24]. The sequences were aligned using CLUSTAL W as implemented in MEGA v.11 software [25]. Maximum likelihood (ML) trees were reconstructed with 1000 bootstrap replicates using MEGA v.11 software. The finalized trees were visualized using in the FigTree v.1.4.2 program [26].

### 2.4. Serological Investigation

All serum samples were tested using commercially available blocking enzyme-linked immunosorbent assays (bELISA), following manufacturer’s instructions (INgezim^®^ West Nile Compac, Ingenasa, Madrid, Spain). In doing so, species-independent detection of WNV and USUV antibodies against domain III of the coat protein was feasible. Samples were considered positive if the inhibition percentage (IP) was >40%, doubtful with IP ≥30% to ≤40%, and negative with IP <30%.

In parallel, all samples were tested for the presence of USUV- and WNV-specific nAbs with virus neutralization tests (VNTs) using Vero B4 and Vero 76 cells, respectively, under biosafety level 3 conditions as described by Seidowski et al. 2010 [27]. WNV strain Germany (lineage 2, GenBank accession No. MH924836) and USUV strain Germany (Europe 3, GenBank accession No. HE599647) were used to quantify cross-reacting nAbs among the Japanese encephalitis serogroup. Negative and positive controls (sera negative for flavivirus nAbs and sera from experimentally infected animals or hyperimmune sera from vaccinated animals, respectively) were used to ensure the VNTs’ success.

The nAb titer was calculated according to the Behrens-Kaerber method [28]. Neutralizing antibody titers (ND_50_) were determined as the reciprocal of serum dilution inhibiting >50% of the cytopathogenic effects. Serum samples were considered positive with ND_50_ values ≥ 10 and negative with ND_50_ values < 10 [28]. The nAbs in a serum sample were considered specific against a flavivirus if only one of the viruses was neutralized or there was a fourfold difference between the ND_50_ titers against the two viruses. The result is interpreted as not differentiable if the antibody titers against both viruses are similar. This is the case when cross-reactivity makes it difficult to distinguish between WNV- and USUV-specific nAbs.

### 2.5. Pathology and Immunohistochemistry

Gross examinations were performed of two great grey owls (No. 60 and 68) and representative samples were fixed in 4% neutral buffered formalin, dehydrated, and embedded in paraffin. Sections of 3 µm were prepared and mounted on Superfrost plus slides. Sections of all samples were stained with haematoxylin and eosin for histopathological examination. For immunohistochemistry, an in-house polyclonal antibody (pab U433) was used for the specific detection of USUV antigen. To exclude cross-reactivity an additional section was treated with an in-house pab-detecting WNV antigen (pab OM8). For both antibodies the pre-treatment included rehydration and inhibition of endogenous peroxidase with 3% H_2_O_2_ (Merck, Darmstadt, Germany) in methanol for 30 min. Sections used with pab U433 were treated in citrate buffer (pH 6.1) for 20 min at 600 W in the microwave, whereas sections used with pab OM8 were treated with Proteinase K (Roche, Mannheim, Germany) (with 4 g/mL for 15 min at 37 °C). A serum block was applied in both protocols directly before the incubation with the primary pab (U433 at a dilution of 1:4000 and OM8 at a dilution of 1:1700 in goat serum) for two hours at room temperature. Negative control sections were incubated with goat serum alone. For development, the EnVision reagent (Dako Diagnostics, Hamburg, Germany) and diaminobenzidine-tetrahydrochloride counterstained with Mayer’s haematoxylin were used.

### 2.6. Ethical Statement

The animals were kept at the zoological facility according to European husbandry guidelines and national animal welfare regulations. Residual blood material was available from birds collected primarily for veterinary examination, diagnostic purposes, specific treatments, and determining the effectiveness of a treatment.

## 3. Results

### 3.1. Molecular Results

During the course of this study, no increase in bird mortality was observed. In total, 193 blood and tissue samples of 92 individuals were analyzed, among which USUV-RNA was detected in eight samples (Table 1 and Table 2). Two positive great grey owls (*Strix nebulosa*) (No. 91 and 92) died in the summer of 2018 and tested positive for USUV (Ct-values of 15.32 and 21.26) as described by Michel et al. 2019 [15]. In March 2021, four owls (No. 8, 9, 10, and 49) that were clinically healthy and showed no signs of disease tested positive or doubtful for USUV during routine monitoring. In addition, USUV was detected in two great grey owls (No. 60 and 68) which died peracutely in the summer of the same year (2021). After dissection, USUV was found in the brains, livers, and spleens with Ct-values between 20.08 and 25.73 (Table 2). In December 2021, a Northern hawk-owl (*Surnia ulula*) (No. 27) was found dead but WNV genome could not be detected at any time point. Infections with avian influenza virus could be excluded (data not shown).

### 3.2. Phylogenetic Analyses

**Figure 2:**
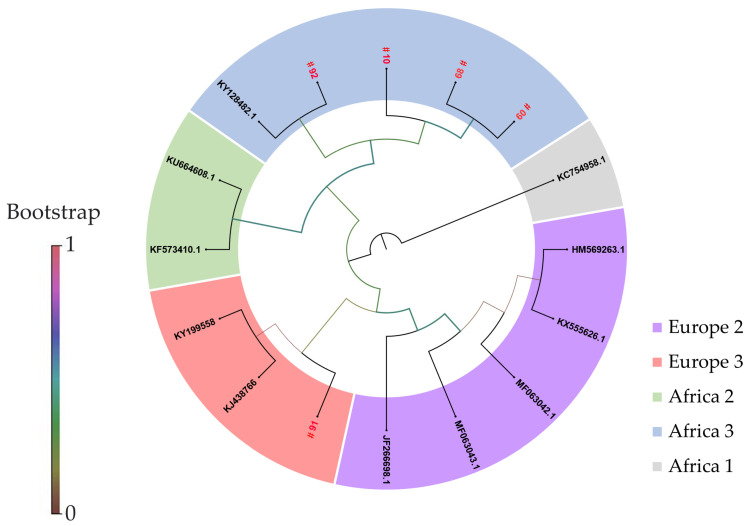
Phylogeny of the sequenced USUV isolates from 2018–2022. Phylogenetic analysis of USUV whole-genome strains detected in birds from Wildpark Schwarze Berge. Sequences are labelled by codes containing the bird’s ID. The sequences from Germany described in this study are highlighted in red. (# Whole genome sequence are already published in Bergmann et al. 2023 [29] (Genbank Accession No.: OQ630904–OQ630908)).

Phylogenetic analyses revealed the distribution of USUV lineage Africa 3 in 2018 and 2021, whereas Europe 3 was only detected once in 2018 in a deceased great grey owl (No. 91) (Table 2). Figure 2 shows the phylogenetic tree of all sequenced birds. Phylogenetic analyses clearly revealed a close relationship between USUV genomes from both great grey owls (No. 60 and 68), which were housed as conspecifics in the same aviary and died within a few days of each other. The same is evident for the Northern long-eared owl (*Asio otus*) (No. 10) which was infected only five months earlier with a USUV strain that clustered close to those from the great grey owls (No. 60 and 68) (Figure 2).

### 3.3. Serological Results

This longitudinal study shows the antibody courses of the sampled animals over several years. A period of four years was chosen to investigate the course of flavivirus infections in different zoo birds. To assess virus circulation in Germany serological results were interpreted together with the molecular results.

#### 3.3.1. Serological Results Obtained by Blocking ELISA (bELISA)

**Figure 3:**
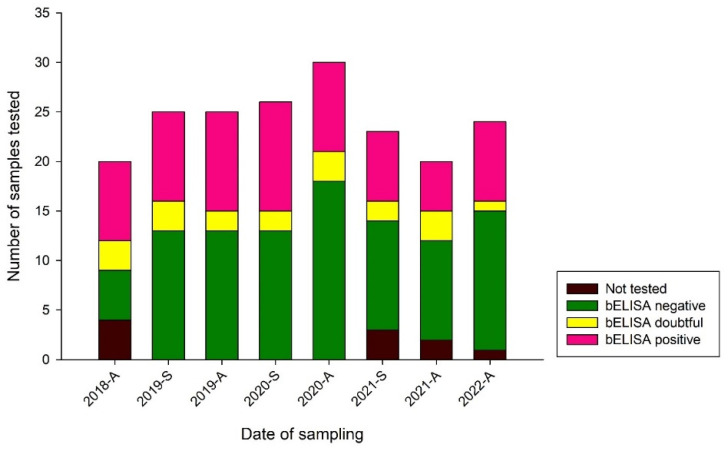
Stacked bars depicting the distribution of blocking ELISA (bELISA) results from 2018 to 2022. Samples for which bELISA screening was not possible are depicted in black. Negative bELISA results are depicted in green, doubtful results in yellow, and positive results in pink. The date of sampling is portrayed per year and season (A = autumn and S = spring).

Flavivirus antibodies were detected with bELISA in 46% (42/92) (positive samples/tested samples) of the individual zoo birds: 27% in *Anseriformes* (3/11), 67% in *Ciconiiformes* (4/6), 0% in *Coraciiformes* (0/1), 27% in *Galliformes* (3/11), 0% in *Gruiformes* (0/1), 57% in *Passeriformes* (4/7), and 49% in *Strigiformes* (72/146). Figure 3 depicts the distribution of the bELISA results for each sampling date with a constant number of bELISA-positive samples during the course of this study.

#### 3.3.2. Serological Results with Virus Neutralization Assays (VNTs)

**Figure 4:**
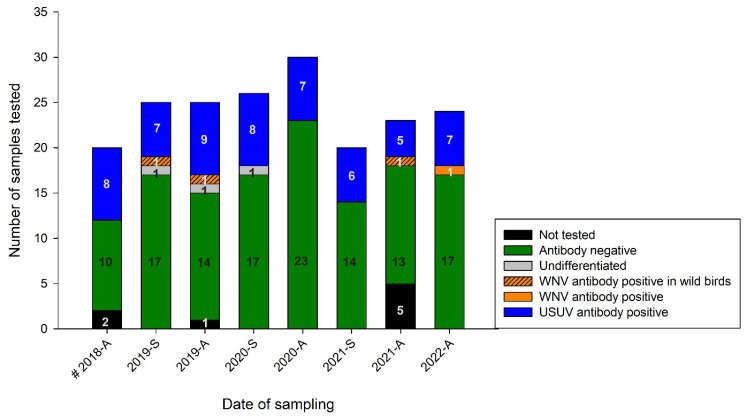
Stacked bars showing the distribution of virus neutralization assay (VNT) results from 2018 to 2022. All investigated VNTs with negative VNT results for USUV/WNV depicted in green, not differentiable results in grey, and USUV-positive results in blue. WNV-antibody-positive samples are shown in orange, including three wild birds (migratory birds) found on the zoo premises shown in orange with patterns. Samples for which no VNTs were performed (due to small sample volumes) are depicted in black and are described as not tested. The date of sampling is given per year and season (A = autumn and S = spring); #: the detailed serological results from the time point “2018-A” were already published in Michel et al. 2019 [15].

All samples were additionally screened by USUV- and WNV-specific VNTs. All negative bELISA results were confirmed with VNT, with the exception of two samples from autumn 2019 that belonged to a Northern long-eared owl (No. 10) (USUV ND_50_ 1/80) and a Eurasian scops owl (No. 15) (USUV ND_50_ 1/15; WNV ND_50_ 1/10). In Figure 4, the distribution of the VNT results throughout the sampling period is depicted. A total of 50 bELISA-reactive serum samples from 20 different individuals were validated with USUV VNT. USUV nAbs in the sera of the birds were already detected upon onset of the sampling in 2018. The WNV VNTs, on the other hand, yielded only three positive samples (No. 23, 32, and 79) originating from wild/migratory birds and only one positive sample from a zoo bird (No. 85). It was surprising, however, that the last sampling at the end of the study in autumn 2022 identified the first zoo bird (golden pheasant (*Chrysolophus pictus*)) hatched in 2022 in Wildpark Schwarze Berge with high nAb titers against WNV (WNV ND_50_ 1/240). A detailed distribution of the USUV and WNV nAbs in all the samples which tested positive for neutralizing antibodies is illustrated in Figure 4 and Table S2.

#### 3.3.3. Serological Results in the Temporal Context of this Longitudinal Study

**Figure 5:**
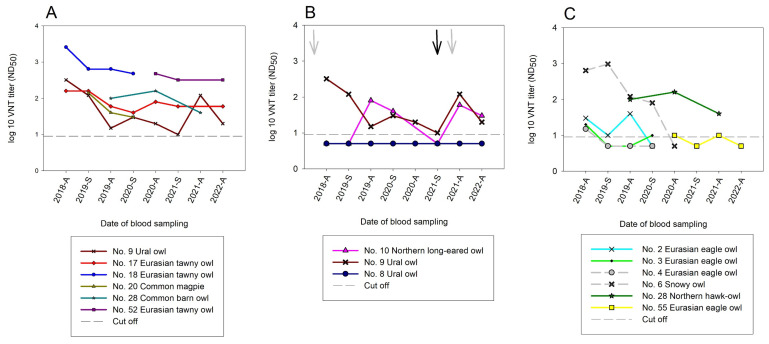
Neutralizing antibodies in zoo birds induced by a USUV infection and detected with virus neutralization test (VNT) during the study period. (**A**) Birds with USUV nAbs throughout the study period; (**B**) Owls tested positive/doubtful for USUV genome during routine monitoring in March 2021 (RT-qPCR values listed in Table 2). Grey arrows indicate the detection of USUV-RNA in the organs of the deceased birds that are not displayed in the figure. The black arrow marks the detection of USUV-RNA in the blood of the illustrated birds; (**C**) Owls with a drop in their nAb titers and suspected to have had a USUV reinfection yet lacking molecular evidence thereof.

In six birds (No. 9, 17, 18, 20, 28, and 52), the development of USUV nAbs could be observed over an extended period of time without the antibody titer falling below the detection limit. However, sampling was not consistently possible for four of the six birds (Figure 5A). On a molecular level, USUV-RNA was detected in two owls in March 2021 (No. 9 and 10) which resulted in an increase in antibody titers. In comparison, a third great grey owl (No. 8) revealed only doubtful USUV RT-qPCR results in 2021 and did not develop nAbs (Figure 5B).

In four birds (No. 2, 4, 6, and 55), this longitudinal study showed a drop in the nAb titers below the detection limit. In addition, a drop followed by an increase in USUV nAbs was observed in six birds (No. 2, 3, 9, 10, 28, and 55) (Figure 5C). On a serological level, this suggests a new introduction of USUV into the population. For example, an increase in USUV nAbs was detected in an eagle owl (*Bubo bubo*) (No. 2) towards the end of 2019, suggesting a reinfection with USUV in the summer of 2019 following a terminated infection (nAbs detected in autumn 2018) (Figure 5C). At the same time, an increase in the nAb titers was detected in the Northern long-eared owl (No. 10) in 2019, as well as in 2021 following a proven reinfection (Figure 5B). The Northern hawk owl (*Surnia ulula*) (No. 28) showed a slight increase in nAb titers in autumn 2020 compared to 2019, suggesting a USUV reinfection in the summer of 2020 (Figure 5C).

Due to natural fluctuations and the purchase or sale of zoo birds, the antibody progression could only be followed in five birds throughout the entire study period of four years. Of these only three birds (No. 9, 10, and 17) had USUV nAbs, while two birds (No. 5 and 8) were antibody-negative (Figure 5A, Tables S2 and S3).

Fifteen seropositive zoo birds were sampled only once: ten birds had USUV nAbs (No. 12, 35, 37, 50, 58, 67, 69, 70, 87, and 88), four birds were detected with WNV nAbs (No. 23, 32, 79, and 85), and for one bird the results were not differentiable (No. 24) (Table S2). Overall, USUV titers between ND_50_ 1/10 and ND_50_ 1/2560 and WNV titers between ND_50_ 1/10 and ND_50_ 1/240 were detected with VNT. The results are summarized in Table S2.

### 3.4. Gross Examination, Histopathology, and Immunohistochemistry

**Figure 6:**
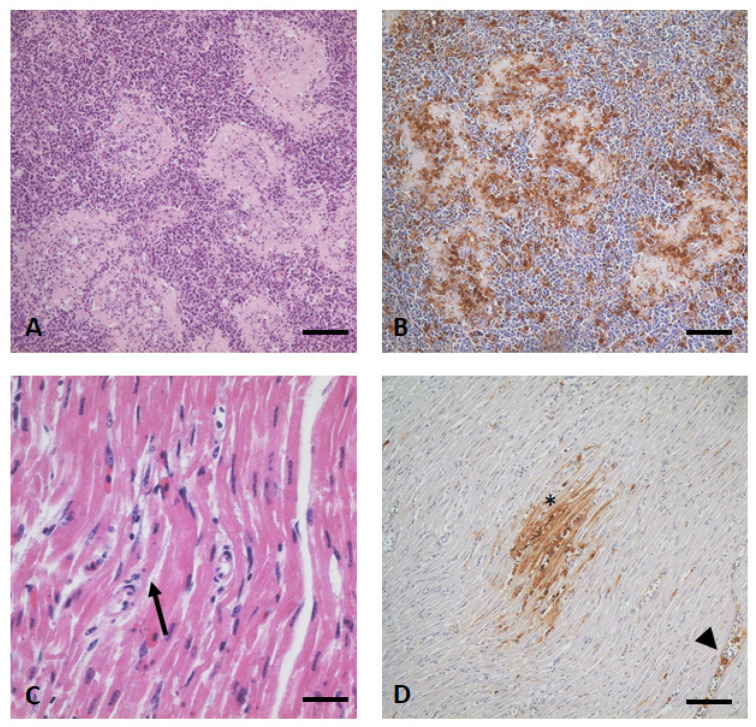
Results from the histopathological and immunohistochemical examination of the great grey owl (No. 68). (**A**) Multifocal fibrinoid necrosis in the sheathed arteries of the spleen with (**B**) distinct deposition of USUV antigen in necrotic foci and mononuclear cells; (**C**) very mild focal necrosis of a muscle fiber in the heart (arrow) and (**D**) moderate accumulation of USUV antigen (asterisk), highlighting the positive reactivity of intraluminal single macrophages in the small vessel in the right corner of the figure (arrowhead); immunohistochemistry, anti-USUV (in-house polyclonal antibody U433), Bar (**A**,**B**,**D**) 50 µm and (**C**) 20 µm.

A male and a female great grey owl were subjected to gross examination. Both animals were in good body shape but showed signs of advanced autolysis and freezing artefacts. Nevertheless, the male (No. 68) revealed a fatty but small spleen, whereas the female (No. 60) showed focal subepicardial hemorrhages, a pale and brittle spleen, as well as a pale and mildly enlarged liver. However, the results of the histopathological examinations were very similar in both birds, with a predominant severe multifocal periarteriolar fibrinoid necrosis of the lymphoid sheaths (Figure 6A) accompanied by mild infiltrations of fatty macrophages and lymphoid depletions. In addition, both birds showed multifocal mild periportal and intralobular foci of single hepatocellular necrosis as well as oligofocal mild foci of necrotizing myocarditis (Figure 6C). The brain was only weakly affected in both birds, with oligofocal signs of mild acute nonsuppurative encephalitis (i.e., lymphohistiocytic perivascular cuffing, glia nodules, astrogliosis, satellitosis) in the cerebellum (No 68) and cerebrum (No. 60 and 68) or mesencephalon (No. 60). The female additionally showed oligofocal a mild acute nonsuppurative vasculitis in the lung and in the large intestine a mild subacute fibrinoid necrotizing enteritis. Multifocal mild deposition of uric acid crystals in the kidney (gout) was an additional incidental finding in both animals.

Both birds showed a widespread distribution of USUV antigen, most prominent in the spleen (Figure 6B), liver, kidney, heart (Figure 6D), lung, and gut (including but less prominent in the proventriculus). The male (No. 68) revealed viral antigen also in the brain, muscle, and pancreas, whereas in the female (No. 60) the ganglion aorticorenale was also affected. Supplemental Table S4 gives a more detailed overview concerning the amount and distribution of the detected USUV lesions in both birds.

## 4. Discussion

Monitoring activities demonstrated the introduction of USUV into Germany in 2010/2011 [12,13]. Zoological gardens constitute an ideal habitat for mosquitoes, for example, due to the existence of many ponds. In combination with the presence of competent vectors and birds as amplifying hosts, zoos fulfil the required conditions for the transmission of Flaviviruses. Once a virus has been introduced into a zoo the risk for local transmissions outside of the facility also increases. The past years have confirmed that zoological facilities are valuable early-warning systems in terms of timely detection of flavivirus outbreaks. In the Vienna Zoo in 2001, a large USUV outbreak was discovered with the death of five great grey owls. In Italy [10] and at the Zurich Zoo [11], widespread deaths of wild and captive birds were observed. In America, one of the first WNV cases was detected at the Bronx Zoo in 1999 [30]. An advantage of the present study is not only the surveillance of zoo birds but also the possibility to monitor them in the frame of a longitudinal study. This study began in 2018, when the first WNV case was discovered in Germany and mass deaths were observed in blackbirds across the country due to USUV infections [14,15]. It ended in 2022, when several WNV cases occurred in close proximity to the zoo [16] (Figure 1).

In the frame of this study, eight birds were found infected with USUV (Table 1 and Table 2), half of which died (No. 91, 92, 60, and 68), while the other four birds (No. 8, 9, 10, and 49) survived the infection without showing clinical signs. Molecular analyses thereby confirmed the circulation of USUV in spring 2021 and the summers of 2018 (No. 91 and 92) and 2021 (No. 60 and 68). Not surprisingly, lower Ct-values were detected in the (organ) samples from the deceased birds (15.32 to 25.73), compared to those that survived the infection (blood samples) with Ct-values ranging from 32.79 to 37.12 (Table 2). However, one must keep in mind that the viral loads in organ samples can in general be higher than in blood samples as demonstrated by Benzarti et al. 2020 [31]. Phylogenetic analyses detected the USUV lineages Africa 3 and Europe 3 in 2018 (Table 2 and Figure 2), confirming their distribution in Lower Saxony [15]. The lineage Africa 3 was detected again in 2021 (No. 10, 60, and 68) (Figure 2) indicating the circulation of this lineage throughout the study period. Recent in vitro and in vivo studies have shown a significant difference in the virulence between different USUV lineages [32,33]. USUV lineage Europe 2 appears to be more neurovirulent in mouse models [32] whereas lineage Africa 3 showed less pathogenic effects and lower levels of viremia in mice [33]. However, the question of transferability from the mouse model to birds has not been sufficiently clarified. Due to the small sample size (92 birds) and the single occurrence of USUV lineage Europe 3 in one bird, no conclusions on the pathogenicity of the different lineages can be drawn from this study.

In neighboring countries (e.g., the Netherlands [34]) and zoological facilities (e.g., Vienna 2001 [8] and Berlin 2015 [35]), an increased mortality was observed in common blackbirds and great grey owls, indicating a high susceptibility of great grey owls to severe and often fatal USUV infections. This is in accordance with the results of the here described longitudinal study, as four out of the five USUV-positive great grey owls (No. 49, 60, 68, 91, and 92) developed a lethal USUV infection. The fifth great grey owl (No. 49) died in the fall of 2021 due its advanced age and not in connection with its USUV infection. Numerous avian species are susceptible to USUV infections but the induced lesions are very similar, aside from individual differences. Among these, the brain, liver, spleen, heart, and kidney are most commonly affected (Refs. [10,34,35,36,37,38], for detailed review see [39]) as seen in the great grey owls of this study. This is also reflected in the immunohistochemical detection of viral antigen in both birds (No 60 and 68), which is often widely distributed in numerous cell types of various organs and tissues [39]. Interestingly, however, no viral antigen was detected in the brain except for in individual macrophages in the vessels. This has already been described by Störk et al. 2021 [38], where USUV antigen was detectable in the brain of only 45% of the infected animals. This finding contrasts with previous reports where up to 90% positive brain samples were found in the studied cohorts [31,36]. This individual variability in USUV tissue distribution must be considered when studying routine specimens.

Unfortunately, this longitudinal study did not have further information on the birds’ origins, making it difficult to interpret the determined positive nAb titers from the start of the study (autumn 2018). Furthermore, the transition of zoo birds from other zoological facilities in other parts of Germany or Europe may have had an impact on the resilience of the immune system to flavivirus infections. These birds may have had in the past contact with other (unknown) arboviruses, which triggered their immune system. However, due to the mass occurrence of USUV in Germany in 2018, it is very likely that the obtained titers at the beginning of this study were a consequence of the infection pressure in the summer. These data are comparable to those for wild birds in this very same region [15].

Previously, USUV nAb titers have only been detected over a time frame of two years in zoo birds [40]. By contrast, the here presented study shows a longitudinal monitoring of zoo birds over a four-year period with the evidence of USUV circulation (Table 2 and Figure 5). For the first time, five birds (No. 5, 8–10, and 17) could be continuously sampled and serologically examined for USUV over four years. Of these birds three (No. 9, 10, and 17) were nAb-positive and two (No. 5 and 8) remained nAb-negative (Figure 5A, Table S3). Additionally, several other birds tested USUV nAb-positive for two and three years, respectively. In this study a direct correlation between the detection of USUV and the increase in nAbs could be shown, therefore easing the way for future interpretations with incomplete data (only serological or molecular evidence). Furthermore, given a certain time interval between infections a booster effect on the nAbs could be observed (Figure 5B). Exemplary hereof was the Northern long-eared owl (No. 10) that showed a decline in nAb titers followed by renewed increase in correlation with a verified acute USUV infection.

Lastly, serological investigations revealed the first introduction of WNV into the zoological garden in 2022 in a juvenile golden pheasant (No. 85) with antibody titers of ND_50_ 1/240. The golden pheasant showed no clinical signs; however, it died only two weeks after sampling. Unfortunately, no necropsy was performed. The circulation of WNV in 2022 was underscored by the detection of WNV-RNA in two wild birds and findings of WNV-IgM antibodies in a horse in close proximity [16]. Additionally, serological studies in raccoons (*Procyon lotor*) and raccoon dogs (*Nyctereutes procyonoides*) revealed the presence of WNV in the western part of Lower Saxony in January 2022 [41]. Since 2019, with the detection of WNV in only one migratory wild bird in Hamburg, these were the first reported WNV cases in this region [16] (Figure 1). In the zoo study, too, neither WNV genome nor WNV nAbs were noted in the zoo birds prior to 2022 (Table 1, Tables S2 and S3).

The detection of WNV in Wildpark Schwarze Berge underlines the importance of zoos as an early-warning system for wild bird surveillance. In addition to the highly susceptible birds sampled here, ducks and geese could also be included in future monitoring programs as they are susceptible to WNV infections, show hardly any clinical signs, and seroconvert rapidly with high nAb titers [42]. Furthermore, zoo mammals have also proven to be a valuable predictor of arbovirus infections [40,43].

## 5. Conclusions

This longitudinal study revealed the detection of USUV-specific nAbs in birds over a four-year period, proving the relevance of long-term monitoring studies in zoo birds (resident birds) in the surveillance of emerging flaviviruses. Especially in urban regions, zoo bird surveys can be a useful early-warning system in detecting the emergence of new pathogens and uncovering their spatial distribution. Detected nAbs appear to result in a good protection from severe disease progression in most birds despite environmental viral pressure, with the exception of highly susceptible great grey owls. To which extent the detected new introduction of WNV in the fourth year of this study has triggered further mortalities will be revealed in future studies.

## Figures and Tables

**Table 1:** Sampled zoo birds listed by species order with USUV and WNV RT-qPCR results as a function of all birds tested. Positive RT-qPCR results are highlighted in bold.

Species Order	Common Name	Scientific Name	USUV Positive/ Birds Tested	WNV Positive/ Birds Tested
*Anseriformes*	Eurasian teal	*Anas crecca*	0/2	0/2
	Canada goose	*Branta canadensis*	0/1	0/1
	Graylag goose	*Anser anser*	0/1	0/1
	Barnacle goose	*Branta leucosis*	0/1	0/1
	Muscovy duck	*Cairina moschata*	0/2	0/2
	Ruddy shelduck	*Tadorna ferruginea*	0/1	0/1
	Mallard	*Anas platyrhyn.*	0/1	0/1
	Lesser white-fronted goose	*Anser erythropus*	0/2	0/2
*Ciconiiformes*	Black stork	*Ciconia nigra*	0/1	0/1
	White stork	*Ciconia ciconia*	0/4	0/4
*Coraciiformes*	Laughing kookaburra	*Dacelo novaeguineae*	0/1	0/1
*Galliformes*	Golden pheasant	*Chrysolophus pictus*	0/3	0/3
	Common pheasant	*Phasianus colchicus*	0/4	0/4
	Hamburg chicken	*Gallus gallus domesticus*	0/2	0/2
*Gruiformes*	Common crane	*Grus grus*	0/1	0/1
*Passeriformes*	Common magpie	*Pica pica*	0/1	0/1
	Common raven	*Corvus corax*	0/3	0/3
*Strigiformes*	Great grey owl	*Strix nebulosa*	**4**/5	0/5
	Ural owl	*Strix uralensis*	**2**/2	0/2
	Common barn owl	*Tyto alba*	**1**/11	0/11
	Snowy owl	*Bubo scandiacus*	0/8	0/8
	Northern hawk-owl	*Surnia ulula*	0/7	0/7
	Eurasian eagle owl	*Bubo bubo*	0/11	0/11
	Eurasian tawny owl	*Strix aluco*	0/7	0/7
	Northern long-eared owl	*Asio otus*	**1**/8	0/8
	Eurasian scops owl	*Otus scops*	0/2	0/2
			**8**/92	0/92

**Table 2:** Zoo birds which tested positive or doubtful for USUV with RT-qPCR. Ct-values, sampling dates and matrices (tissues), USUV lineages, and GenBank accession numbers are given.

Bird’s ID	Species Order	Common Name	Scientific Name	Ct-Value ^¶^	Tissue	Date of Sampling	USUV Lineage	GenBank Accession No.
91 *^,#^	*Strigiformes*	Great grey owl	*Strix nebulosa*	15.75	Brain	08-2018	Europe 3	OQ630904
				15.32	Liver			
92 *^,#^	*Strigiformes*	Great grey owl	*Strix nebulosa*	21.26	Brain	09-2018	Africa 3	OQ630905
				18.30	Liver			
8	*Strigiformes*	Ural owl	*Strix uralensis*	37.12	Blood coagulum	03-2021	nt	nt
9	*Strigiformes*	Ural owl	*Strix uralensis*	33.23	Blood coagulum	03-2021	nt	nt
10 ^#^	*Strigiformes*	Northern long-eared owl	*Asio otus*	32.79	Blood coagulum	03-2021	Africa 3	OQ630908
49	*Strigiformes*	Common barn owl	*Tyto alba*	35.77	Blood coagulum	03-2021	nt	nt
60 ^#^	*Strigiformes*	Great grey owl	*Strix nebulosa*	23.88	Brain	08-2021	Africa 3	OQ630907
				20.48	Liver			
				22.44	Spleen			
68 ^#^	*Strigiformes*	Great grey owl	*Strix nebulosa*	25.73	Brain	08-2021	Africa 3	OQ630906
				20.08	Liver			
				21.47	Spleen			

* Partial sequences previously published in Michel et al. 2019 [15] (Accession No.: OQ630904– OQ630908); ^#^ Whole genome sequences published in Bergmann et al. 2023 [29]; nt, not tested; ^¶^ Ct-values were determined according to a protocol described by Cavrini et al. 2011 [18].

## Data Availability

The data that supports the findings of this study are available in the main manuscript and the Supplementary Materials of this article.

## Supplementary Materials

The following supporting information can be downloaded at https://www.mdpi.com/article/10.3390/pathogens12060753/s1. Table S1: Characteristics of all zoo birds tested during the study period indicating all sampling dates; Table S2: bELISA, WNV and USUV neutralization assay results in all birds tested positive; Table S3: bELISA, WNV and USUV neutralization assay results in all birds tested negative; Table S4: Amount and distribution of USUV antigen in both great grey owls (No. 60 and 68) as shown with immunohistochemistry (in house polyclonal antibody U433).
